# A pilot study on the differential urine proteomic profile of subjects with community-acquired acute kidney injury who recover versus those who do not recover completely at 4 months after hospital discharge

**DOI:** 10.3389/fmed.2024.1412561

**Published:** 2024-08-19

**Authors:** Harpreet Kaur, Kajal Kamboj, Sachin Naik, Vivek Kumar, Ashok Kumar Yadav

**Affiliations:** 1 Department of Experimental Medicine & Biotechnology, Postgraduate Institute of Medical Education and Research, Chandigarh, India; 2 Department of Nephrology, Postgraduate Institute of Medical Education and Research, Chandigarh, India

**Keywords:** community-acquired acute kidney injury, proteomic, renal outcome, LC–MS/MS, CKD

## Abstract

**Background:**

Community-acquired acute kidney injury (CA-AKI) is a sudden structural damage and loss of kidney function in otherwise healthy individuals outside of hospital settings having high morbidity and mortality rates worldwide. Long-term sequelae of AKI involve an associated risk of progression to chronic kidney disease (CKD). Serum creatinine (SCr), the currently used clinical parameter for diagnosing AKI, varies greatly with age, gender, diet, and muscle mass. In the present study, we investigated the difference in urinary proteomic profile of subjects that recovered (R) and incompletely recovered (IR) from CA-AKI, 4 months after hospital discharge.

**Methods:**

Study subjects were recruited from ongoing study of CA-AKI cohort. Patients with either sex or age > 18 years with no underline CKD were enrolled at the time of hospital discharge. Incomplete recovery from CA-AKI was defined as eGFR < 60 mL/min/1.73 m2 or dialysis dependence at 4 months after discharge. Second-morning urine samples were collected, and proteome analysis was performed with LC–MS/MS. Data were analyzed by Proteome Discoverer platform 2.2 (Thermo Scientific) using statistical and various bioinformatics tools for abundance of protein, cellular component, protein class and biological process were analyzed in the recovered and incompletely recovered groups.

**Results:**

A total of 28 subjects (14 in each group) were enrolled. Collectively, 2019 peptides and proteins with 30 high-abundance proteins in the incompletely recovered group (R/IR <0.5, abundance ratio adj. *p*-value <0.05) and 11 high-abundance proteins in the incompletely recovered group (R/IR >2.0, abundance ratio adj. *p*-value <0.05) were identified. Tissue specificity analysis, GO enrichment analysis, and pathway enrichment analysis revealed significant proteins in both the groups that are part of different pathways and might be playing crucial role in renal recovery during the 4-month span after hospital discharge.

**Conclusion:**

In conclusion, this study helped in identifying potential proteins and associated pathways that are either upregulated or downregulated at the time of hospital discharge in incompletely recovered CA-AKI patients that can be further investigated to check for their exact role in the disease progression or repair.

## Introduction

Acute kidney injury (AKI) is a complex disorder with a sudden decrement in kidney function, involving structural damage and loss of kidney function. Since it has mixed etiologies that include glomerulonephritis, interstitial nephritis, nephrotoxicity, ischemia, and sepsis, this syndrome lacks distinct pathophysiology (1, 2). The causes of AKI in low-and middle-income countries vary from those in high-income countries. In high-income countries, sepsis, multiorgan failure, and toxic effects from drugs seem to be one of the most common causes. However, in low-and middle-income countries (LMICs), it is more frequently seen in young ones, and the situation is worsened by incidences of malaria, HIV/AIDS, and dengue, but in rural areas of LMIC, AKI is generally community-acquired instead of hospital-acquired, accompanied by suboptimal antenatal care, poor regulation of indigenous medical practices, consumption of unclean water, diarrhea, so on (3, 4). CA-AKI mostly occurs in one’s community and household area due to infections, malaria, diarrheal diseases, without any previous hospital contact, and mostly in subjects which are otherwise healthy.

Renal damage due to AKI is not ephemeral. AKI provides a ground for the subsequent progression into chronic kidney disease (CKD). The body’s own instinct and effector mechanisms to protect itself from any disease do follow after AKI. Nevertheless, certain factors such as cellular stress, genetic stress, old age, and pre-existing comorbidities such as hypertension and diabetes, render the AKI affected individual susceptible to maladaptive repair of damaged renal cells, eventually leading to CKD (5–7). Hence, efforts are being made by researchers to facilitate early prediction of AKI progression to CKD. The widely used criteria for determining AKI are by measuring serum creatinine (SCr) levels, urine output, and glomerular filtration rate (GFR). Unfortunately, these parameters are often misleading since they fluctuate with age, gender, muscle mass, and diet which decreases the accuracy of the kidney health assessment (8, 9). Many other urinary and serum biomarkers predicting AKI outcomes have been found, for instance, neutrophil gelatinase-associated lipocalin (NGAL), L-type fatty acid-binding protein (L-FABP), kidney injury molecule-1 (KIM-1), tissue inhibitor of metalloproteinases *2* (TIMP-2), insulin-like growth factor-binding protein 7 (IGFBP7), cystatin C, netrin-1, and uromodulin (UMOD) (10–12). Various biomarkers for AKI incidence and progression to CKD using proteomics have been identified including exosomal fetuin-A (13, 14), zinc-*α*-2-glycoprotein (15), aprotinin (16), *α*-1 microglobulin (15, 17, 18), *α*-1 acid glycoprotein (15), albumin (15), *β*-2 microglobulin (17, 19), hepcidin-25 (20, 21), and uromodulin (12), although TIMP-2 and IGFBP7 are the only two FDA-approved biomarkers in the US and Europe (22).

CA-AKI is quite divergent in terms of epidemiology and clinical profile from HA-AKI. So, biomarkers are being evaluated for both types of AKI that occur in different settings. Despite the substantial research in the understanding of AKI to CKD progression, the identification of potential biomarkers that can predict CA-AKI to CKD transition and reduce or halt the impact of progressing CKD is still under investigation. There is a knowledge gap in why some patients do not recover completely even when their baseline characteristics have reached safe levels as those who recover completely after hospital discharge. In the present study, we aim to identify the proteins that are differentially expressed at the time of hospital discharge in patients with CA-AKI who do not recover completely as compared to those who recover completely at 4 months after hospital discharge for their utilization as predictive biomarkers for AKI to CKD development.

## Materials and methods

### Study population

The study subjects were enrolled from the observational study of CA-AKI cohort at PGIMER, Chandigarh, India, between February 2017 and January 2020 (23). All subjects admitted at PGIMER, Chandigarh, and between the age of 18 and 70years who were diagnosed with AKI at any time during hospital stay and discharged from hospital were eligible for screening. Patients were categorized under CA-AKI only if the subject developed AKI at the time of hospital admission within 48h of initial contact with healthcare services. Subjects were not enrolled in the study if they were solid organ or bone marrow transplant recipients, suspected diagnosed case of heart failure with functional class 3 or 4 or high likelihood of underlying CKD as determined by treating physician based on clinical and laboratory parameters and nephropathy as the cause of AKI and patients with pre-existing CKD with baseline CKD EPI eGFR<60 mL/min/1.73m^2^ prior to onset of illness. Written informed consent was obtained from each patient, and the study was approved from the Institute Ethic committee of PGIMER, Chandigarh. All procedures were performed in accordance with the Declaration of Helsinki guidelines.

### Study conduct and follow-up

Baseline characteristics, clinical and laboratory data including detailed urine analysis, and demographic details of the patients were collected after enrollment and at the time of discharge. Second-morning midstream urine samples were collected at the time of hospital discharge and processed by low-speed centrifugation for 10 min, 2000 × g at room temperature. The supernatant was collected and stored at −80°C for proteomic analysis. The subjects had scheduled follow-up visits at 1 and 4 months after discharge. All clinical events occurring in enrolled subjects and laboratory parameters were documented at every follow-up visit.

### Recovery and incomplete recovery from CA-AKI

Renal recovery was defined as eGFR (CKD EPI-2009) > 60 mL/min/1.73 m^2^ and spot urine protein-to-creatinine ratio (uPCR) < 500 mg/g at 4 month after hospital discharge. Patients with eGFR<60 mL/min/1.73 m^2^ or dialysis dependent at 4 month after discharge were defined as incomplete renal recovery.

### Proteomic urine sample preparation

Urine samples from CA-AKI patients were subjected to preparation for proteomic analysis by modifying the method from Court et al. (24). The proteins in the 1 mL urine sample were precipitated, solubilized, reduced, and alkylated. Protein concentration was measured using bicinchoninic acid protein assay (BCA) and proceeded with 200 μg protein concentration for trypsin digestion. Next day, 10% TFA (pH 2) was added in samples to stop the reaction. Samples were resuspended in an adequate volume of 1% TFA v/v and 5% acetonitrile (ACN) to reach a final concentration of 1 μg/μl followed by dilution with loading buffer (0.2% formic acid v/v, 2% ACN v/v).

### LC–MS/MS analysis

The urine samples were then analyzed in Thermo Scientific™ Orbitrap Fusion™ Tribrid™ mass spectrometer (LC–MS/MS). The PicoFrit analytical column (75 μm × 25 cm, 5 μm BetaBasic C18, 150 Å, New Objective, MA) was used for liquid chromatography. The column to which processed urine samples was added was washed several times with different concentrations of ACN and formic acid solution followed by elution of the bounded proteins in the “flow”. Sample volume was adjusted according to the sample protein concentration of 1–2 μg/μl. Following the separation, the column was equilibrated, and survey scans were acquired at a resolution of 70,000 over a mass range of m/z 250 to m/z 1,800 with automatic gain control (AGC) target of 10^6^. The raw files containing spectrums and graphs acquired by the MS system were processed using the Proteome Discoverer platform 2.2 (Thermo Scientific) and viewed by Xcalibur™ Software. Peptides and proteins quantified by LC–MS/MS along with their accession number, abundance in each sample, abundance ratio (recovered/incompletely recovered), abundance ratio adjusted *p*-value, FDR (False Discovery rate), and *q*-value were acquired.

### Protein shortlisting

The peptides and proteins obtained after subjecting urine samples to LC–MS/MS were shortlisted by >2-fold change in protein expression: downregulated in the R group (R/IR <0.5, abundance ratio adj. *p*-value <0.05) and upregulated in the R group (R/IR >2, abundance ratio adj. p-value <0.05). Proteins with abundance ratio of <0.01 and > 40.0 were excluded to avoid false positives. Selected proteins in the two groups were then compared for tissue specificity using Bgee,^1^ for Gene Ontology (GO) cellular component, protein class, and biological process using Panther,^2^ and for pathway enrichment using Reactome Knowledgebase^3^ and Panther (25) to identify the peptides with significant different levels in the two groups.

## Statistical analyses

Statistical analyses were performed using SPSS version 23. Baseline characteristics and clinical parameters of patients at the time of hospital discharge and 1 and 4 months after hospital discharge were compared using Mann–Whitney U-test. Categorical data were expressed as numbers (percentages) and compared using the chi-square test. Numerical variables were expressed as mean ± standard deviation (SD). All tests were performed to compare different parameters between the completely recovered (R) and incompletely recovered (IR) groups. A two tailed *p*-value of <0.05 was considered statistically significant.

## Results

### Baseline characteristics of study subjects

We enrolled 28 randomly selected CA-AKI patients (14 recovered and 14 incompletely recovered CA-AKI) from ongoing observational study. Patient characteristics and clinical parameters are shown in Tables 1, 2. The mean age was 44.45 ± 17.40 years with 55% of the enrolled patients being female. All patients enrolled in this study were in stage 3 AKI. During the admission, a total of 19 (68%) patients required dialysis, including 8 (57%) in the recovered group and 11 (78%) in the incompletely recovered group. A total of 22 (79%) were oliguric at the time of admission (10 in the recovered group and 12 in the incompletely recovered group). The mean serum creatinine at admission and discharge was 7.33 ± 3.60 mg/dL and 3.17 ± 1.78 mg/dL, respectively. Estimated glomerular filtration rate (eGFR) was 61.67 ± 46.49 mL/min/1.73 m^2^ in recovered patients and 14.22 ± 8.77 mL/min/1.73 m^2^ in incompletely recovered patients at the time of discharge. There was no significant difference in the groups in baseline characteristics and biochemical parameters except serum creatinine at discharge (*p* = 0.009), eGFR (*p* = 0.008), and blood urea (*p* = 0.010) (Table 1). The primary cause of CA-AKI in enrolled patients was sepsis (55%), followed by ischemia (20%), nephrotoxins (15%), and multifactorial causes (10%).

The IR group at 1-month and 4-month follow-ups displayed higher serum creatinine (*p* = 0.005 and *p* = 0.005, respectively) and lower eGFR (*p* = 0.005 and *p* = 0.001, respectively) with more improvement in the recovered group (Table 2). Similarly, urine protein-to-creatinine ratio (uPCR) was markedly higher in the IR group at 4-month follow-up (*p* = 0.023), highlighting possible kidney damage.

### Generation of urinary protein profile and protein shortlisting

**FIGURE 1:**
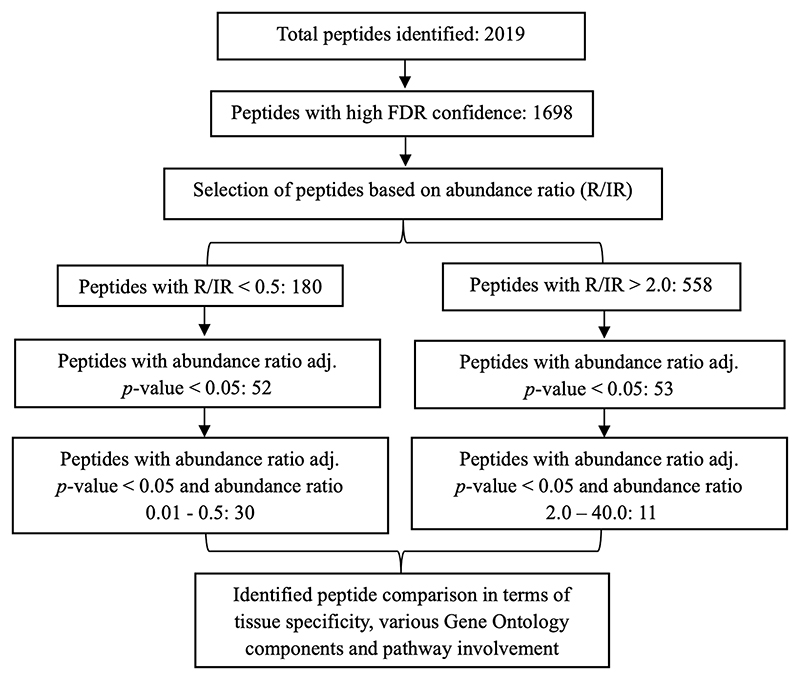
Schematic representation of the shortlisting of proteins differentially expressed in recovered and incompletely recovered CA-AKI subjects.

**FIGURE 2:**
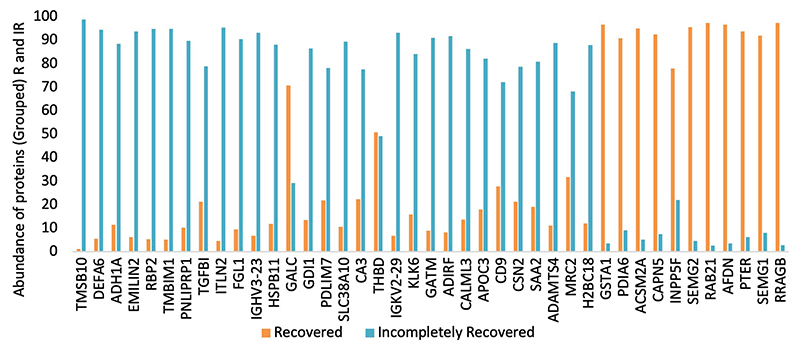
Urinary protein abundance in the recovered (R) and incompletely recovered (IR) groups.

In total, 2019 peptides and proteins were obtained collectively in both groups after subjecting the processed urine samples to LC–MS/MS. Figure 1 depicts the protein shortlisting steps. To identify the differentially upregulated proteins in the two groups, proteins were divided based on R/IR abundance ratio of <0.5 (proteins downregulated in the R group) and > 2.0 (proteins upregulated in the R group). A total of 52 proteins in the R/IR <0.5 group and 53 proteins in the R/IR >2.0 group with significant difference (*p* < 0.05) were selected for further shortlisting (Supplementary Tables S1, S2), and 30 downregulated proteins (R/IR = 0.01–0.5, adjusted *p*-value of <0.05) and 11 upregulated proteins (R/IR = 2–40.0, adjusted *p*-value of <0.05) in the R group were shortlisted. The proteome of selected 30 and 11 proteins differs markedly between the two groups as shown in Figure 2.

### Determining tissue specificity of selected urinary proteome

Table 3 demonstrates the presence of high-abundance proteins in both the groups in different tissues in the form of Bgee expression score. Proteins with high levels in the IR subjects are widely expressed all over the body and do not exhibit a trend. In total, 8 out of 30 proteins exhibited high expression in adult mammalian kidney as indicated by Bgee expression score of more than 90.0 along with high expression in other tissues and organs such as GI tract mucosa, pancreas, liver, rectum, cerebellum, ovary, oocyte, uterine tube, artery, prostate gland, renal medulla, and parts of respiratory tract.

Most of the proteins upregulated in R subjects express highly in urinary excretory organs and tissues including ureters, urinary bladder, kidney renal medulla, and renal glomerulus. It also includes male and female reproductive organs such as cervix, uterus, and testis. In total, 4 out of 11 proteins have an expression score of more than 90.0 in adult mammalian kidney along with high expressivity in other body tissues and organs including liver, kidney, excretory system, male and female reproductive system, adrenal tissue, pancreas rectum, heart right ventricle, and adrenal tissue (Table 3). SEMG1 is not expressed in the kidney but is majorly expressed in the male reproductive system.

### Go enrichment analysis of selected proteome

**FIGURE 3:**
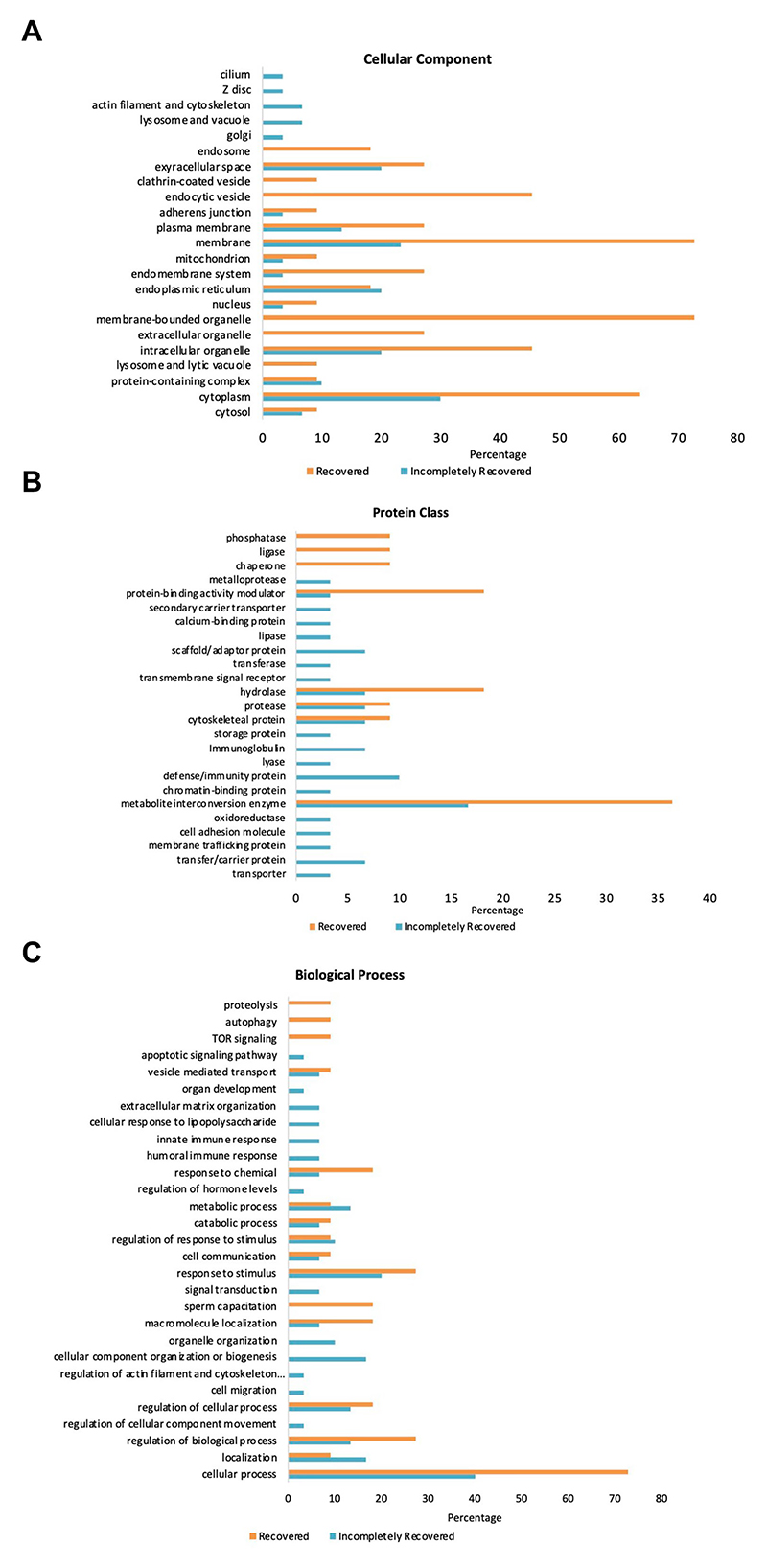
GO Panther analysis of differentially expressed proteins in the R and IR groups. **(A)** Cellular component. **(B)** Protein class. **(C)** Biological process.

Figure 3 illustrates GO-slim enrichment analysis of the urinary selected proteome in the R and IR groups. The cellular component of GO-slim analysis indicates that most of the selected proteins are excreted from the extracellular space, plasma membrane, membrane, endoplasmic reticulum, intracellular organelle, and cytoplasm with all the proteins being excreted more in the R/IR >2.0 group except for the endoplasmic reticulum proteins. In addition, proteins excreted from endocytic vesicles and membrane-bounded organelles were only present in the R group (Figure 3A).

Furthermore, the PANTHER protein class in GO analysis of the 30 and 11 proteins of the two groups demonstrated that a significant number of proteins belonging to hydrolase, protease, cytoskeletal protein, and metabolite interconversion enzyme class are present in urine samples of both R and IR patients. However, the IR group has some proteins belonging to scaffold/adaptor protein, immunoglobulin, defense/immunity protein, and transfer/carrier protein class that are present in high abundance and are unique to their urine samples. Similarly, the number of proteins in protein-binding activity modulator, hydrolase, and metabolite interconversion enzyme class is significantly higher in urine samples collected from recovered individuals (Figure 3B).

PANTHER GO-slim enrichment of the biological process depicts that most of the shortlisted proteins are involved in response to chemical, response to stimulus, sperm capacitation, macromolecule localization, regulation of cellular process and biological process, localization, and cellular process. Proteins involved in apoptotic signaling pathway, innate and humoral immune response, and cellular response to lipopolysaccharide are unique to urine samples of IR subjects. On the other hand, response to chemical, macromolecule localization, regulation of biological process, and cellular process were the processes upregulated in the R group (Figure 3C). SAA2 in the IR group was not found in the GO enrichment analysis.

### Pathway enrichment analysis of urinary proteome

Involvement of the selected proteins in various pathways was observed using Reactome. For the IR group, the Reactome Knowledgebase generated signal cascades for 23 out of 30 shortlisted proteins, where 122 pathways were hit by at least one of the 30 proteins (Supplementary Figure S1). Supplementary Table S3 enlists the 31 most significant pathways identified in the R/IR <0.5 group (*p* < 0.05, FDR <0.1) using Reactome (25). For the R group, the Reactome Knowledgebase generated signal cascades for 9 out of 11 shortlisted proteins, where 61 pathways were hit by at least one of the 11 entered proteins (Supplementary Figure S2). Supplementary Table S4 enlists the 21 most significant pathways identified in the R/IR >2.0 group (*p* < 0.05, FDR <0.12).

**FIGURE 4:**
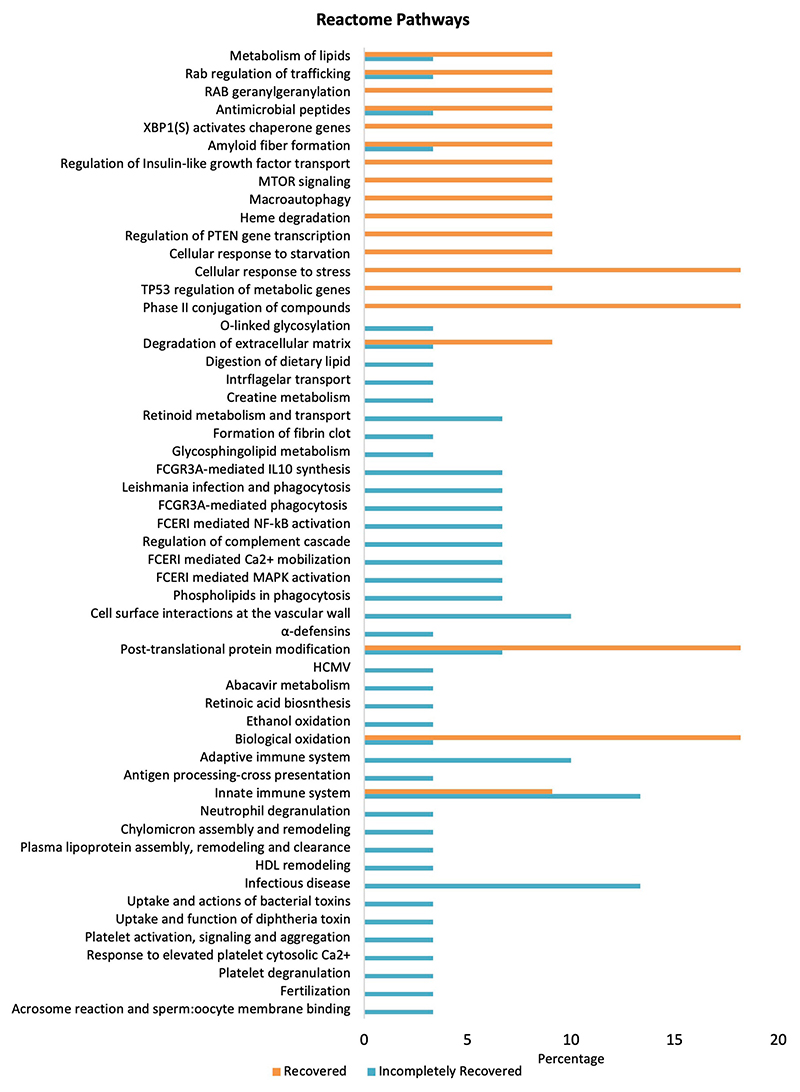
Pathway enrichment analysis differentially expressed proteins in the R and IR groups (*p* < 0.05) using Reactome pathway in PANTHER.

Furthermore, Reactome pathways were investigated using the PANTHER database to identify significantly upregulated pathways in which the high-abundance proteins of both the groups are involved (Figure 4). Proteins in the IR group significantly upregulate pathways including cell surface interactions at the vascular wall, adaptive immune system, innate immune system, and infectious disease pathways compared to the R group. However, cellular response to stress, phase II conjugation of compounds, post-translational protein modification, and biological oxidation-related pathways are downregulated in the IR group. Moreover, fibrin clot formation/fibrosis, a hallmark of CKD, was only present in the IR group.

## Discussion

In our study, we assessed proteins via LC–MS/MS in the urine of the patients at the time of discharge that either recovered completely or incompletely from CA-AKI at 4 months after hospital discharge. The differential urine proteomic profile of R and IR subjects highlighted significant differences in abundance of various proteins, their cellular component, protein class, biological process, and pathway involvement which might be useful in identifying a protein panel that are upregulated or downregulated in patients at the time of hospital discharge and may predict the recovery or non-recovery from CA-AKI. This information will help predict CA-AKI progression to CKD and help the clinician for better management providing appropriate drug regimens. In a recent study, proteinuria and kidney function were found to be correlated with urinary peptidome in healthy and CKD patients (26).

Our study identified 30 and 11 proteins of high abundance in the urine samples of the IR and R groups. The upregulated proteins in the IR subjects did not exhibit any specific tissue specificity, while proteins with high levels in the urine samples of the R group were majorly expressed in excretory organs and tissues with high expression in male and female reproductive organs, liver, heart, and adrenal tissue as well. Almost a similar number of proteins located in extracellular space were upregulated in R and IR subjects. This indicates enhanced glomerular permeability and might also represent early CKD pathology (27, 28) which is justified since the CA-AKI patients were not fully recovered when discharged from the hospital. Similarly, a high abundance of proteins functioning in endoplasmic reticulum indicates high unfolded protein response and ER stress (29) in both the groups. A similar number of proteins located in mitochondrion were upregulated in both groups, indicative of regulation of mitochondrial dynamics after an episode of damage caused by AKI (30). Maximum number of proteins in both the groups fall under metabolite interconversion enzyme class which coincides with the fact that a healing body involves converting or degrading various non-desired products with the aim of normal renal function (31).

PANTHER GO-slim enrichment of the biological process and pathway enrichment analysis via Reactome highlighted significant differences between the upregulated pathways in the two groups. Increased levels of proteins participating in apoptotic signaling (TMBIM1), innate (TMBIM1, IGKV2-29, IGHV3-23, and DEFA6), humoral immune response (MRC2, IGKV2-29, and IGHV3-23), and cellular response to lipopolysaccharides (IGHV3-23 and DEFA6) represent the enhanced mitochondrial pathway of apoptosis which might contribute toward AKI to CKD progression. Lipopolysaccharide is a well-known cause of sepsis-associated AKI that induces tubular cell apoptosis characterized by mitochondrial membrane depolarization, cytochrome c release, and cell shrinkage indicative of enhanced mitochondrial pathway of apoptosis and increasing cytokine release (32, 33) which also explains the reason for the heightened immune response against the endotoxin. Apoptotic renal tubular cells increase renal inflammation, acting as a major source of proinflammatory cytokines and chemokine release and thus contributing to increased immune response (34) in the IR group. NF-κB activation in renal tubular and interstitial cells after AKI episode exacerbates renal health (35), which is further supported by heightened FCERI-mediated NF-κB activation (IGKV2-29, IGHV3-23) in the IR group. Moreover, the presence of the formation of fibrin clot GO biological process only in IR is indicative of fibrosis, a common CKD pathological condition (36) as indicated by higher TGFB1 levels (37). However, high THBD levels that play a crucial role in regulating inflammation and maintaining vascular homeostasis, especially during heightened intravascular coagulation after endothelial injury, indicate injury repair (38) which demonstrates that the body is still in transitioning mode to recovery at the time of hospital discharge. Proteins involved in the regulation of complement cascade (IGKV2-29 and IGHV3-23) and upregulated in the IR group aggravate AKI and its advancement to various kidney diseases (26). In a recent study, MRC2 has been found to be playing an important role in diabetic nephropathy as its level increases in kidneys but its knockdown promotes cellular apoptosis showing its role as potential biomarker for diabetic nephropathy (39).

Although prolonged autophagy might cause kidney damage, few studies have shown the reno-protective role of autophagy as it aids in renal tubular cell regeneration and repair (40, 41). Similarly, enhanced mTOR signaling in renal tubules promotes post-AKI renal regeneration (42, 43). Thus, enhanced RRAGB levels, autophagy, and TOR signaling protein in the R group explain renal recovery. Phase II conjugation enzymes including transferases that promote detoxification and easy renal excretion of compounds were upregulated in the R group. Levels of some transferases increase during sepsis but are non-predictive of AKI incidence (44). Markedly, high levels of proteins participating in cellular response to stress (RRAGB and PDIA6) and regulation of biological (AFDN, INPP5F, and RRAGB) and cellular processes (SEMG1, SEMG2, RAB21, ACSM2A, INPP5F, RRAGB, PDIA6, and GSTA1) in R subjects might be due to enhanced organelle stress response including organelles such as ER, mitochondria, and cilia leading to activation of various signaling pathways to provide nephroprotection and regain homeostatic conditions (45). Mitochondrial *β*-oxidation is significantly impaired in AKI making mitochondria dysfunctional along with promoting renal fibrosis, thus favoring AKI to CKD progression (46–48). Thus, enhanced biological oxidation-related pathways in R individuals as indicated by upregulated ACSM2A and GSTA1 might explain kidney reverting to normal functioning at the time of hospital discharge. Upregulated post-translational protein modification in the R group as indicated by high abundance of PDIA6 indicates renal recovery post-kidney insult since PDI inhibition leads to renal cell apoptosis and disturbs mitochondrial homeostasis (49).

Recent studies have identified and proposed various candidate biomarkers which have been divided into three major categories: stress markers (demonstrate cellular stress that might result in kidney damage), damage markers (reflecting structural damage that might result in kidney damage), and functional markers (indicating kidney dysfunction and altered GFR) (39, 50, 51). Thus, kidney-damaging/incomplete recovery markers IGKV2-29 and IGHV3-23 aggravate renal damage. Moreover, TMBIM1, DEFA6, MRC2, IGKV2-29, and IGHV3-23 can possibly be classified under renal stress markers as they promote renal inflammation and, hence, renal injury including MRC2 which like other markers promotes cellular apoptosis enhancing kidney damage. On the contrary, renal recovery markers RRAGB and GSTA1 were enhanced in R individuals. In addition, PDIA6, AFDN, INPP5F, SEMG2, ACSM2A, RAB21, and GSTA1 were the proteins upregulated in the R group and involved in stress response and so can be classified as renal recovery stress markers possibly exhibiting neuroprotective roles by mediating cellular stress caused by AKI.

The study has its strength as it was conducted in well-characterized and phenotyped patients with CA-AKI. However, there are few limitations: It was a pilot study conducted on small sample size and absence of validation of differentially expressed protein in the recovered and incompletely recovered groups.

In conclusion, this study helped in identifying potential proteins and associated pathways that are either upregulated or downregulated at the time of hospital discharge in incompletely recovered CA-AKI patients that can be further investigated to check for their exact role in the disease progression or repair. Although these proteins were not validated and need to be studied in a larger population cohort of CA-AKI for their potential use as biomarker for renal outcome after CA-AKI, the data from this study may help in advancement of our understanding of proteins/pathways associated with repair mechanism in CA-AKI.

## Supplementary Material

Figures and tables The Supplementary material for this article can be found online at: https://www.frontiersin.org/articles/10.3389/fmed.2024.1412561/full#supplementary-material

## Figures and Tables

**Table 1:** Baseline characteristics and clinical parameters of CA-AKI subjects at the time of hospital discharge.

Characteristics	Total no. of patients (*n* = 28)	Recovered (*n* = 14)	Incompletely recovered (*n* = 14)	*p*-value
Age in years	44.450 ± 17.40	38.6 ± 17.92	50.30 ± 15.563	0.137
Female sex (%)	15 (53.57%)	8 (57.14%)	7 (50.00%)	0.707
SBP (mm/Hg)	116.60 ± 14.21	119.20 ± 14.39	114.00 ± 14.29	0.428
DBP (mm/Hg)	74.700 ± 8.80	74.40 ± 9.55	75.00 ± 8.49	0.884
Requirement of RRT (%)	19 (67.85%)	8 (57.14%)	11 (78.57%)	0.224
SCr at admission (mg/dl)	7.33 ± 3.60	6.81 ± 3.13	7.85 ± 4.12	0.532
SCr at discharge (mg/dl)	3.17 ± 1.78	2.19 ± 1.65	4.16 ± 1.34	0.009
eGFR (ml/min/1.73 m^2^)	37.94 ± 40.61	61.67 ± 46.49	14.22 ± 8.77	0.008
Hb (g/dl)	9.90 ± 2.21	9.97 ± 2.29	9.822 ± 2.2775	0.887
TLC	8,109 ± 4702.16	8541.11 ± 3983.20	7678.74 ± 5540.78	0.710
Platelets (10*3)	303.88 ± 140.83	266.44 ± 147.75	341.33 ± 131.01	0.272
Blood urea (mg/dl)	68.19 ± 47.42	41.01 ± 19.04	95.37 ± 52.47	0.010
Total bilirubin (mg/dl)	1.06 ± 1.13	0.675 ± 0.21	1.50 ± 1.57	0.136
Direct bilirubin (mg/dl)	0.40 ± 0.50	0.21 ± 0.11	0.597 ± 0.65	0.101
SGOT (U/L)	37.92 ± 25.99	36.80 ± 18.84	39.05 ± 32.82	0.861
SGPT (U/L)	36.37 ± 25.12	42.36 ± 32.53	30.38 ± 14.21	0.326
ALP (U/L)	169.12 ± 143.47	142.14 ± 63.39	196.10 ± 195.16	0.442
Total Protein (g/dl)	7.05 ± 1.2	7.15 ± 1.35	6.960 ± 1.23	0.760
Serum albumin (g/dl)	3.37 ± 0.61	3.48 ± 0.61	3.26 ± 0.633	0.465
Uric acid (mg/dl)	8.32 ± 2.72	7.19 ± 1.89	9.62 ± 3.06	0.082

Continuous variables are expressed as mean ± SD or number (percentage); SBP, systolic blood pressure; DBP, diastolic blood pressure; RRT, renal replacement therapy; SCr, serum creatinine; eGFR, estimated glomerular filtration rate; Hb, hemoglobin; TLC, total leukocyte count; SGOT, serum glutamic oxaloacetic transaminase; SGPT, serum glutamic pyruvic transaminase; ALP, alkaline phosphatase.

**Table 2:** Clinical parameters of CA-AKI subjects at 1-and 4-month follow-ups.

Characteristics	Total no. of patient (*n* = 28)	Recovered (*n = 14*)	Incompletely recovered (*n* = 14)	*p*-value
***At 1 month after discharge***				
SCr (mg/dl)	2.22 ± 1.29	1.00 ± 0.23	1.56 ± 0.47	0.005
eGFR (ml/min/1.73 m^2^)	118.85 ± 65.39	82.28 ± 25.41	50.19 ± 17.95	0.005
uPCR	0.92 ± 0.28	0.18 ± 0.09	0.35 ± 0.27	0.178
***At 4 month after discharge***				
SCr (mg/dl)	2.20 ± 1.16	0.89 ± 0.19	1.44 ± 0.50	0.005
eGFR (ml/min/1.73 m^2^)	113.44 ± 74.62	94.32 ± 18.99	54.92 ± 22.65	0.001
uPCR	0.75 ± 0.34	0.24 ± 0.10	0.46 ± 0.22	0.023

Continuous variables are expressed as mean ± SD or number (percentage); SCr, serum creatinine; eGFR, estimated glomerular filtration rate; uPCR, urine protein creatinine ratio.

**Table 3:** Tissue specificity of 30 downregulated protein in (R/IR <0.5) and 11 upregulated protein in the R group (R/IR >2).

Protein name (Urinary proteome)	MW (kDa)	Tissue specificity	Expression score	Expression score for adult mammalian kidney
***R/IR<0.5***				
Thymosin beta-10 (TMSB10)	5	Cortical plate, monocyte, mucosa of transverse colon, granulocyte, ganglionic eminence	99.98, 99.96, 99.96, 99.95, 99.95	99.46
Glycine amidinotransferase, mitochondrial (GATM)	48.4	pancreas, liver, jejunal mucosa, corpus callosum, renal medulla	99.74, 99.48, 99.47, 99.2, 99.2	99.14
CD9 antigen (CD9)	25.4	Pharyngeal mucosa, seminal vesicle, synovial joint, urethra, penis	99.83, 99.8, 99.72, 99.72, 99.59	98.29
Adipogenesis regulatory factor (ADIRF)	7.9	Left and right coronary artery, adipose tissue, ascending aorta, prostate gland,left uterine tube	99.77, 99.69, 99.66, 99.28, 99.27	97.69
Protein lifeguard 3 (TMBIM1)	34.6	Lower esophagus mucosa, descending thoracic aorta, ascending aorta, pancreas, right lung, ectocervix	99.41, 99.14, 99.08, 99.07, 98.92, 98.49	96.36
Rab GDP dissociation inhibitor alpha (GDI1)	50.6	right hemisphere of cerebellum, right frontal lobe, ganglionic eminence, ovary, uterine tube	99.71, 99.63, 99.47,98.87, 98.59	95.51
Putative sodium-coupled neutral amino acid transporter 10 (SLC38A10)	119.7	adenohypophysis, pancreas, liver, right uterine tube, right coronary artery	97.93, 97.67, 97.47, 95.90, 95.85	91.2
Intraflagellar transport protein 25 homolog (HSPB11)	16.3	Oocyte, bronchial epithelial cell, nasopharynx, right uterine tube, pancreatic ductal cell	99.58, 99.48, 98.32, 97.79, 97.57	90.02
PDZ and LIM domain protein 7 (PDLIM7)	49.8	Uterus, right coronary artery, lower esophagus muscularis layer, left uterine tube, endocervix	99.64, 99.61, 99.54, 99.52, 99.07	84.33
Galactocerebrosidase (GALC)	77	Adrenal tissue, bronchial epithelial tissue, jejunal mucosa, renal glomerulus, metanephric glomerulus	98.79, 96.74, 96.45, 96.09, 96.03	83.81
Transforming growth factor-beta- induced protein ig-h3 (TGFBI)	74.6	Granulocyte, monocyte, stromal cell of endometrium, ascending aorta, spleen	99.08, 98.51, 98.27, 96.77, 96.43	83.52
C-type mannose receptor 2 (MRC2)	166.6	Tendon of biceps brachii, endometrium, pericardium, right ovary, urethra	99.6, 99.18, 97.68, 96.96, 96.86	83.29
Calmodulin-like protein 3 (CALML3)	16.9	Lower esophagus mucosa, tongue squamous epithelium, upper arm skin, leg and abdomen skin, cervix epithelium	99.33, 98.73, 98.47, 96.93, 96.29	77.87
Thrombomodulin (THBD)	60.3	Gingival epithelium, vena cava, right lung, skin of hip and abdomen, left uterine tube, mucosa of urinary bladder	96.2, 94.98, 94.75, 94.18, 92.83	75.54
Apolipoprotein C-III (APOC3)	10.8	Jejunal mucosa, right lobe of liver, amniotic fluid, metanephros, left uterine tube	99.98, 99.96, 74.89, 66.28, 65.31	74.32
Kallikrein-6 (KLK6)	26.8	Spinal cord, inferior vagus X ganglion, corpus callosum, dorsal motor nucleus of vagus nerve, renal glomerulus	99.38, 98.21, 96.02, 93.33, 90.05	73.66
Histone H2B type 2-F (H2BC18)	13.9	Bone marrow cell, male germ line stem cell, adrenal tissue, blood, corpus callosum	98.0, 93.07, 92.69, 87.79, 85.68	67.03
Elastin microfibril interface-located protein 2 (EMILIN2)	115.6	Decidua, monocyte, stromal cell of endometrium, granulocyte, left uterine tube	98.84, 96.32, 94.58, 94.42, 88.59	66.31
Serum amyloid A-2 protein (SAA2)	13.5	Right lobe of liver, saliva secreting gland, thoracic mammary gland, left uterine tube, gall bladder	98.41, 96.19, 93.46, 84.38, 79.74	63.48
Carbonic anhydrase 3 (CA3)	29.3	Rectus abdominis, vastus lateralis, diaphragm, tongue, left uterine tube	99.89, 99.69, 99.40,98.33,81.92	62.49
Retinol-binding protein 2 (RBP2)	15.7	Intestinal mucosa, duodenum, buccal mucosa cell, right lung, right uterine tube	99.98, 97.48, 83.09, 75.35, 72.99	61.74
Alcohol dehydrogenase 1A (ADH1A)	39.8	Right lobe of liver, male germ line stem cell, descending thoracic aorta, gall bladder, right uterine tube	99.86, 85.15, 76.80, 76.46, 69.77	54.65
Inactive pancreatic lipase-related protein 1 (PNLIPRP1)	51.8	Pancreas, islets of Langerhans, buccal mucosa cell, primordial germ cell in gonad, ectocervix	99.92, 96.57, 73.77, 72.58, 63.42	49.27
Fibrinogen-like protein 1 (FGL1)	36.4	Liver, pancreas, sperm, endometrium, ectocervix	99.67, 99.05, 90.86, 76.70, 56.93	48.4
Intelectin-2 (ITLN2)	36.2	duodenum, small intestine Peyer’s patch, lungs, coronary artery, rectum	98.78, 86.77, 85.80, 76.75, 54.38	40.68
Defensin-6 (DEFA6)	11	Duodenum, small intestine Peyer’s patch, rectum, mucosa of transverse colon, vermiform appendix	99.95, 96.65, 69.52, 61.31, 59.67	33.59
Immunoglobulin heavy variable 3–23 (IGHV3-23)	12.6	Male germ line stem cell in testis, rectum, duodenum, vermiform appendix, spleen	97.35, 90.07, 89.52, 87.46, 78.07	29.72
Immunoglobulin kappa variable 2–29 (IGKV2-29)	13.1	Male germ line stem cell in testis, lymph node, spleen, vermiform appendix, granulocyte	83.34, 81.33, 80.83, 80.02, 71.02	27.43
Beta-casein (CSN2)	25.4	Male germ line stem cell, tonsil, saliva secreting gland, placenta, urinary bladder	98.48, 48.77, 46.44, 33.81, 30.10	21.1
A disintegrin and metalloproteinase with thrombospondin motifs 4 (ADAMTS4)	90.1			Not found
***R/IR>2***				
Glutathione S-transferase A1 (GSTA1)	25.6	Bronchial epithelial cell, jejunal mucosa, adrenal tissue, renal glomerulus, right testis, left testis	99.86, 99.78, 99.52, 98.56, 98.55, 98.02	97.86
Protein disulfide-isomerase A6 (PDIA6)	48.1	Corpus epididymis,adrenal tissue, islet of Langerhans, germinal epithelium of ovary, rectum	99.62, 99.2, 99.19, 98.72, 98.70	97.23
Acyl-coenzyme A synthetase ACSM2A, mitochondrial (ACSM2A)	64.2	Right lobe of liver, cortex of kidney, metanephros cortex, metanephric glomerulus, male germ line stem cell, mucosa of stomach	99.12, 83.52, 83.29, 76.91, 76.85, 55.45	97.15
Ras-related protein Rab-21 (RAB21)	24.3	Rectus abdominis, heart right ventricle, adrenal tissue, amniotic fluid, rectum, uterus	96.52, 96.37, 95.63, 95.29, 93.06, 90.71	90.63
Afadin (AFDN)	206.7	Right uterine tube, lower esophagus mucosa, bronchial epithelial cell, minor salivary gland, rectum	99.38, 98.70, 98.45, 97.82, 97.61	88.16
Phosphotriesterase-related protein (PTER)	39	Anterior cingulate cortex, corpus epididymis, nephron tubule, renal medulla calcaneal tendon	92.44, 89.37, 88.02, 87.95, 87.43	86.39
Calpain-5 (CAPN5)	73.1	Mucosa of transverse colon, rectum, gall bladder, stromal cell of endometrium, stomach, left testis	96.23, 94.93, 94.21, 91.03, 90.27, 87.40	80.33
Phosphatidylinositide phosphatase SAC2 (INPP5F)	128.3	Pons, superior vestibular nucleus, secondary oocyte, parietal lobe, rectum, uterus	99.71, 99.52, 98.7, 98.52, 81.15, 85.5	78.3
Ras-related GTP-binding protein B (RRAGB)	43.2	Calcaneal tendon, left ovary, right ovary, cortical plate, right uterine tube, ectocervix	94.33, 92.67, 92.40, 92.34, 91.74, 91.36	77.5
Semenogelin-2 (SEMG2)	52.1	Seminal vesicle, sperm, male germ line stem cell, prostate gland, metanephros, metanephros cortex	100, 96.69, 91.32, 53.48, 48.75, 46.1	46.14
Semenogelin-1 (SEMG1)	52.1	Seminal vesicle, sperm, prostate gland, gall bladder, colonic epithelium, thymus	100, 99.61, 62.38, 54.3, 52.96, 50.27	Not found

## Data Availability

The datasets presented in this study can be found in online repositories at Massive.ucsd.edu with Accession No: MSV000095489.

## Footnotes

1
https://www.bgee.org/

2
http://pantherdb.org/

3
https://reactome.org/
