# Supplementary material for: A pilot study on the differential urine proteomic profile of subjects with community-acquired acute kidney injury who recover versus those who do not recover completely at 4 months after hospital discharge
Source: Front Med (Lausanne). 2024 Aug 19;11:1412561. doi: 10.3389/fmed.2024.1412561 (PMC7616407; doi:10.3389/fmed.2024.1412561)
Supplement: Supplementary file 1 [file Data_Sheet_1.PDF]

Supplementary Material

Pilot Study on Differential Urine Proteomic Profile of Subjects with Community Acquired – Acute Kidney Injury who Recover Versus those who Do Not Recover Completely at 4 months after Hospital Discharge

Harpreet Kaur[1,3], Kajal Kamboj[2], Sachin Naik[2], Vivek Kumar[2], Ashok Kumar Yadav[1]

[1]Department of Experimental Medicine & Biotechnology, Postgraduate Institute of Medical Education and Research, Chandigarh, India

[2]Department of Nephrology, Postgraduate Institute of Medical Education and Research, Chandigarh, India

[3]Department of Biochemistry & Biophysics, Texas A&M University, College Station, TX, United States

Table S1: Proteins with abundance ratio of <0.5 and *p* <0.05

Accession number	Description	Abundance Ratio: (Recovered / Incompletely recovered)	Abundance Ratio Adj. *p*-Value: (Recovered / Incompletely recovered)
P37840	Alpha-synuclein	0.01	1.72E-16
P35475	Alpha-L-iduronidase	0.01	1.72E-16
Q8N357	Solute carrier family 35 member F6	0.01	1.72E-16
Q9UKX2	Myosin-2	0.01	1.72E-16
P16401	Histone H1.5	0.01	1.72E-16
Q9Y287	Integral membrane protein 2B	0.01	1.72E-16
P00488	Coagulation factor XIII A chain	0.01	1.72E-16
P15374	Ubiquitin carboxyl-terminal hydrolase isozyme L3	0.01	1.72E-16
P31937	3-hydroxyisobutyrate dehydrogenase, mitochondrial	0.01	1.72E-16
P08118	Beta-microseminoprotein	0.01	1.72E-16
P35080	Profilin-2	0.01	1.72E-16
O15394	Neural cell adhesion molecule 2	0.01	1.72E-16
Q96EE4	Coiled-coil domain-containing protein 126	0.01	1.72E-16
Q96QR1	Secretoglobin family 3A member 1	0.01	1.72E-16
Q00013	55 kDa erythrocyte membrane protein	0.01	1.72E-16
Q15366	Poly(rC)-binding protein 2	0.01	1.72E-16
P38117	Electron transfer flavoprotein subunit beta	0.01	1.72E-16
P08865	40S ribosomal protein SA	0.01	1.72E-16
P00533	Epidermal growth factor receptor	0.01	1.72E-16
A0A0C4DH39	Immunoglobulin heavy variable 1-58	0.01	1.72E-16
P46783	40S ribosomal protein S10	0.01	1.72E-16
P58335	Anthrax toxin receptor 2	0.01	1.72E-16
P63313	Thymosin beta-10	0.012	1.11E-11
Q01524	Defensin-6	0.053	2.1E-06
P07327	Alcohol dehydrogenase 1A	0.048	1.65E-05
Q9BXX0	EMILIN-2	0.058	3.04E-05
P50120	Retinol-binding protein 2	0.067	3.58E-05
Q969X1	Protein lifeguard 3	0.032	8.62E-05
P54315	Inactive pancreatic lipase-related protein 1	0.113	0.000245
Q15582	Transforming growth factor-beta-induced protein ig-h3	0.048	0.00032
Q8WWU7	Intelectin-2	0.081	0.000549
Q08830	Fibrinogen-like protein 1	0.104	0.000646
P01764	Immunoglobulin heavy variable 3-23	0.127	0.001281
Q9Y547	Intraflagellar transport protein 25 homolog	0.134	0.001979
P54803	Galactocerebrosidase	0.062	0.003204
P31150	Rab GDP dissociation inhibitor alpha	0.122	0.003635

Q9NR12	PDZ and LIM domain protein 7	0.19	0.008036
Q9HBR0	Putative sodium-coupled neutral amino acid transporter 10	0.114	0.008991
P07451	Carbonic anhydrase 3	0.204	0.011622
P07204	Thrombomodulin	0.17	0.014958
A2NJV5	Immunoglobulin kappa variable 2-29	0.221	0.015389
Q92876	Kallikrein-6	0.17	0.020917
P50440	Glycine amidinotransferase, mitochondrial	0.11	0.024211
Q15847	Adipogenesis regulatory factor	0.194	0.030329
P27482	Calmodulin-like protein 3	0.159	0.035897
P02656	Apolipoprotein C-III	0.252	0.035897
P21926	CD9 antigen	0.256	0.037527
P05814	Beta-casein	0.199	0.039103
P0DJI9	Serum amyloid A-2 protein	0.26	0.041501
O75173	A disintegrin and metalloproteinase with thrombospondin motifs 4	0.125	0.047048
Q9UBG0	C-type mannose receptor 2	0.25	0.047312
Q5QNW6	Histone H2B type 2-F	0.215	0.047651

Table S2: Proteins with abundance ratio of >2 and p <0.05

**Accession**	**Description**	**Abundance Ratio: (Recovered / Incompletely recovered**	**Abundance Ratio Adj. *p*-Value: (Recovered / Incompletely recovered)**
P08263	Glutathione S-transferase A1	8.003	0.033456
Q15084	Protein disulfide-isomerase A6	10.077	0.023505
Q08AH3	Acyl-coenzyme A synthetase ACSM2A, mitochondrial	11.702	0.023268
O15484	Calpain-5	12.231	0.045477
Q9Y2H2	Phosphatidylinositide phosphatase SAC2	12.616	0.011988
Q02383	Semenogelin-2	19.078	0.030329
Q9UL25	Ras-related protein Rab-21	20.294	0.013797
P55196	Afadin	20.936	0.000166
Q96BW5	Phosphotriesterase-related protein	22.144	0.000332
P04279	Semenogelin-1	33.075	0.004752
Q5VZM2	Ras-related GTP-binding protein B	36.371	0.00051
P41159	Leptin	85.579	1.28E-08
P01854	Immunoglobulin heavy constant epsilon	91.473	1.11E-11
P62820	Ras-related protein Rab-1A	100	1.72E-16
Q9NZZ3	Charged multivesicular body protein 5	100	1.72E-16
P15313	V-type proton ATPase subunit B, kidney isoform	100	1.72E-16
Q99832	T-complex protein 1 subunit eta	100	1.72E-16
P25789	Proteasome subunit alpha type-4	100	1.72E-16
P55259	Pancreatic secretory granule membrane major glycoprotein GP2	100	1.72E-16
O14980	Exportin-1	100	1.72E-16
P39687	Acidic leucine-rich nuclear phosphoprotein 32 family member A	100	1.72E-16
Q9NZ53	Podocalyxin-like protein 2	100	1.72E-16
P52434	DNA-directed RNA polymerases I, II, and III subunit RPABC3	100	1.72E-16
Q9NY33	Dipeptidyl peptidase 3	100	1.72E-16
Q16539	Mitogen-activated protein kinase 14	100	1.72E-16
O00526	Uroplakin-2	100	1.72E-16
Q9Y696	Chloride intracellular channel protein 4	100	1.72E-16
Q9NYL9	Tropomodulin-3	100	1.72E-16
O43598	2'-deoxynucleoside 5'-phosphate N-hydrolase 1	100	1.72E-16
P61018	Ras-related protein Rab-4B	100	1.72E-16
P31040	Succinate dehydrogenase [ubiquinone] flavoprotein subunit, mitochondrial	100	1.72E-16
Q92688	Acidic leucine-rich nuclear phosphoprotein 32 family member B	100	1.72E-16
O75995	SAM and SH3 domain-containing protein 3	100	1.72E-16

Q8TCT8	Signal peptide peptidase-like 2A	100	1.72E-16
P40306	Proteasome subunit beta type-10	100	1.72E-16
P29218	Inositol monophosphatase 1	100	1.72E-16
Q4G0F5	Vacuolar protein sorting-associated protein 26B	100	1.72E-16
Q9HC35	Echinoderm microtubule-associated protein-like 4	100	1.72E-16
P61077	Ubiquitin-conjugating enzyme E2 D3	100	1.72E-16
P16050	Polyunsaturated fatty acid lipoxygenase ALOX15	100	1.72E-16
P18827	Syndecan-1	100	1.72E-16
P80370	Protein delta homolog 1	100	1.72E-16
P07954	Fumarate hydratase, mitochondrial	100	1.72E-16
P49773	Adenosine 5'-monophosphoramidase HINT1	100	1.72E-16
Q02252	Methylmalonate-semialdehyde dehydrogenase [acylating], mitochondrial	100	1.72E-16
O94832	Unconventional myosin-Id	100	1.72E-16
P42566	Epidermal growth factor receptor substrate 15	100	1.72E-16
Q13617	Cullin-2	100	1.72E-16
P49591	Serine--tRNA ligase, cytoplasmic	100	1.72E-16
Q8IUI8	Cytokine receptor-like factor 3	100	1.72E-16
Q15369	Elongin-C	100	1.72E-16
Q9H3R2	Mucin-13	100	1.72E-16
Q9UI12	V-type proton ATPase subunit H	100	1.72E-16

Table S3: Reactome pathway analysis of the proteins selected in R/IR <0.5 group showing most significant pathways with $p < 0.05$

Pathway name	Proteins identified	Total proteins	*p*-value	FDR
Retinoid metabolism and transport	2	44	0.006	0.116
Metabolism of fat-soluble vitamins	2	48	0.007	0.116
Abacavir metabolism	1	4	0.01	0.116
CD22 mediated BCR regulation	2	70	0.014	0.116
Acrosome Reaction and Sperm:Oocyte Membrane Binding	1	6	0.015	0.116
Uptake and function of diptheria toxin	1	7	0.018	0.116
Digestion of dietary lipid	1	7	0.018	0.116
Abacavir ADME	1	9	0.023	0.116
Classical antibody-mediated complement activation	2	95	0.025	0.116
Antigen activates BCR leading to generation of second messengers	2	95	0.025	0.116
Cell surface interactions at the vascular wall	3	246	0.025	0.116
Chylomicron remodeling	1	10	0.025	0.116
Alpha-defensins	1	10	0.025	0.116
Chylomicron assembly	1	10	0.025	0.116
Creatine metabolism	1	10	0.025	0.116
Scavenging of heme from plasma	2	99	0.027	0.116
Visual phototransduction	2	100	0.027	0.116
FCGR activation	2	101	0.028	0.116
HDL remodeling	1	11	0.028	0.116
Role of LAT2/NTAL/LAB on calcium mobilization	2	102	0.028	0.116
Creation of C4 and C2 activators	2	103	0.029	0.116
Reversible hydration of carbon dioxide	1	12	0.03	0.116
Ethanol oxidation	1	12	0.03	0.116
Initial triggering of complement	2	111	0.033	0.116
Role of phospholipids in phagocytosis	2	114	0.035	0.116
FCERI mediated Ca^{2+} mobilization	2	117	0.037	0.116
FCERI mediated MAPK activation	2	119	0.037	0.116
FCGR3A-mediated IL10 synthesis	2	128	0.043	0.116
Binding and uptake of ligands by scavenger receptors	2	129	0.044	0.116
Regulation of complement cascade	2	135	0.147	0.116
Plasma lipoprotein assembly	1	19	0.048	0.116

Table S4: Reactome pathway analysis of the proteins selected in R/IR >2 group showing most significant pathways with p <0.05

Pathway name	Proteins identified	Total proteins	*p*-value	FDR
Phase II-Conjugation of compounds	2	222	0.005	0.083
Drug ADME	2	111	0.005	0.083
Conjugation of salicylate with glycine	1	8	0.008	0.083
Amino Acid conjugation	1	9	0.008	0.083
Conjugation of carboxylic acids	1	9	0.008	0.083
Heme degradation	1	16	0.015	0.083
Synthesis of PIPs at the early endosome membrane	1	16	0.015	0.083
NFE2L2 regulating anti-oxidant/detoxification enzymes	1	17	0.016	0.083
Biological oxidations	2	223	0.018	0.083
Azathioprine ADME	1	23	0.021	0.083
mTORC1-mediated signaling	1	24	0.022	0.083
Energy dependent regulation of mTOR by LKB1-AMPK	1	29	0.027	0.083
Metabolism of porphyrins	1	29	0.027	0.083
Adherens junctions interactions	1	33	0.031	0.083
Cellular responses to stress	3	769	0.032	0.083
Cellular responses to stimuli	3	783	0.033	0.083
Glutathione conjugation	1	37	0.034	0.083
MTOR signaling	1	41	0.038	0.083
Aspirin ADME	1	44	0.041	0.083
XBP1(S) activates chaperone genes	1	48	0.044	0.083
IRE1α activates chaperones	1	50	0.046	0.083

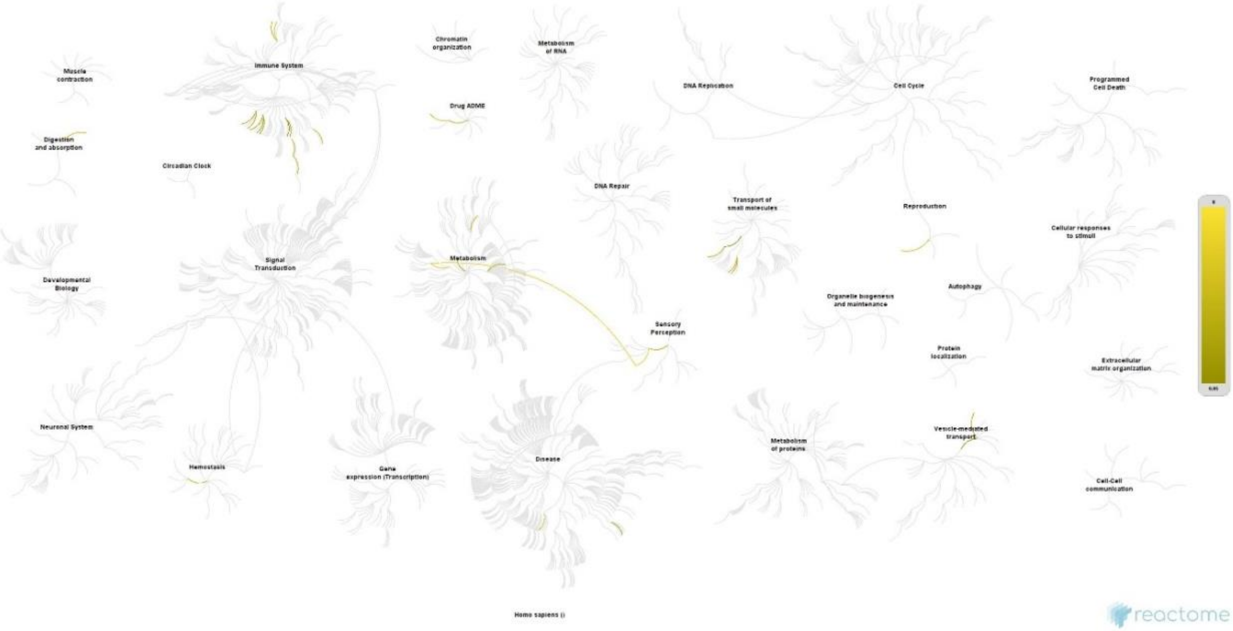

Figure S1: Reactome Knowledgebase pathway overview of the 23 out of 30 proteins in R/IR <0.5 group. Yellow color denotes the enrichment of the respective pathways with a high significance level (*p*-value <0.05)

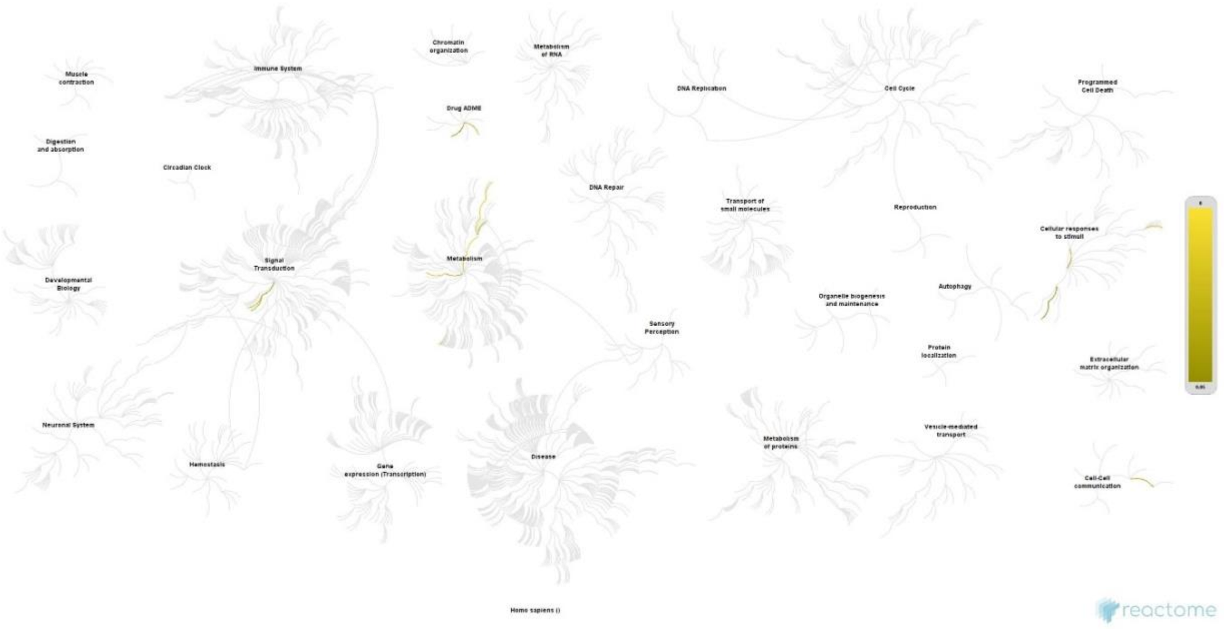

Figure S2: Reactome Knowledgebase pathway overview of the 9 out of 11 proteins in R/IR >2 group. Yellow color denotes the enrichment of the respective pathways with a high significance level (*p*-value <0.05)
